# Dominant Expression of DCLK1 in Human Pancreatic Cancer Stem Cells Accelerates Tumor Invasion and Metastasis

**DOI:** 10.1371/journal.pone.0146564

**Published:** 2016-01-14

**Authors:** Hiromitsu Ito, Shinji Tanaka, Yoshimitsu Akiyama, Shu Shimada, Rama Adikrisna, Satoshi Matsumura, Arihiro Aihara, Yusuke Mitsunori, Daisuke Ban, Takanori Ochiai, Atsushi Kudo, Shigeki Arii, Shoji Yamaoka, Minoru Tanabe

**Affiliations:** 1 Department of Molecular Oncology, Graduate School of Medicine, Tokyo Medical and Dental University, Tokyo, Japan; 2 Department of Hepato-Biliary-Pancreatic Surgery, Graduate School of Medicine, Tokyo Medical and Dental University, Tokyo, Japan; 3 Department of Molecular Virology, Graduate School of Medicine, Tokyo Medical and Dental University, Tokyo, Japan; Peking University Cancer Hospital & Institute, CHINA

## Abstract

Patients with pancreatic cancer typically develop tumor invasion and metastasis in the early stage. These malignant behaviors might be originated from cancer stem cells (CSCs), but the responsible target is less known about invisible CSCs especially for invasion and metastasis. We previously examined the proteasome activity of CSCs and constructed a real-time visualization system for human pancreatic CSCs. In the present study, we found that CSCs were highly metastatic and dominantly localized at the invading tumor margins in a liver metastasis model. Microarray and siRNA screening assays showed that doublecortin-like kinase 1 (DCLK1) was predominantly expressed with histone modification in pancreatic CSCs with invasive and metastatic potential. Overexpression of DCLK1 led to amoeboid morphology, which promotes the migration of pancreatic cancer cells. Knockdown of DCLK1 profoundly suppressed in vivo liver metastasis of pancreatic CSCs. Clinically, DCLK1 was overexpressed in the metastatic tumors in patients with pancreatic cancer. Our studies revealed that DCLK1 is essential for the invasive and metastatic properties of CSCs and may be a promising epigenetic and therapeutic target in human pancreatic cancer.

## Introduction

Pancreatic cancer is the most lethal common cancer because it is usually diagnosed at an advanced stage and is resistant to therapy [1]. The overall survival rate 5 years after diagnosis is approximately 5–6%, which is the lowest rate of any cancer [2]. Despite newly devised surgical techniques and anti-cancer drugs, the treatment efficacy for pancreatic cancer has not significantly improved over the past decade due to the tendency for early invasion and metastasis [3]. These highly malignant characteristics are due to the self-renewal and differentiation of a small subpopulation of cancer cells with stem-like properties, so-called cancer stem cells (CSCs) [4, 5], which are also referred to as metastatic stem cells [6, 7]. Recent studies revealed that the stem cell fate is determined by epigenetic mechanisms including histone modification in either normal cells [8] or CSCs [9]. More importantly, identification of molecular targets of CSCs is expected to accelerate development of novel targeted therapies [10]. Although several cell surface markers have been identified as characteristic of pancreatic CSCs [5, 11], therapeutic targets of the invasive and metastatic process are still unclear in pancreatic cancer [12, 13]. Evaluating therapeutic strategies to target CSCs is difficult because of the complexity of reconstructing mixed populations with differentiated progeny in a hierarchical manner [14, 15]. A monitoring system based on CSC-specific functions could be one solution to these difficulties, and we utilized the low proteasome activity of CSCs to create such a system. Human breast and glioma cancer cells were engineered to stably express green fluorescence fused to the degron of ornithine decarboxylase (Gdeg), which resulted in intracellular accumulation of green fluorescent protein Gdeg as a consequence of the low activity of the 26S proteasome [16, 17]. By using this property, we previously constructed a real-time visualization system for human liver CSCs and demonstrated their high metastatic ability with niche formation [18]. Our visualization system was also used to clarify the highly malignant characteristics of human pancreatic CSCs [19]. In the present study, we identified doublecortin-like kinase 1 (DCLK1) as a protein that is predominantly expressed in invasive and metastatic CSCs. The gene was associated with epigenetics changes including H3K4me3 and H3K27me3 histone modification. DCLK1 was previously reported to be a candidate normal stem cell marker in the gut [20, 21]. However, Nakanishi *et al*. used lineage-tracing experiments and showed that DCLK1 marks tumor stem cells that continuously produce tumor progeny [22]. In addition, Westphalen *et al*. used the same strategy and reported the relationship between long-lived DCLK1-positive cells and initiation of colon cancer [23]. According to the recent report by Bailey *et al*., pancreatic neoplasms in mice that express DCLK1 contain morphologically and functionally distinct subpopulations with CSC-like properties [24]. Using the above visualization system and metastatic pancreatic cancers, our studies revealed that DCLK1 is highly expressed in human pancreatic CSCs and in clinical samples and may represent a therapeutic target.

## Materials and Methods

### Retroviral transduction of the degron reporter into human pancreatic cancer cells

The degron sequence of ornithine decarboxylase (ODC) is recognized directly by proteasomes, which leads to the immediate destruction of the involved protein [16]. The retroviral expression vector pQCXIN-ZsGreen-cODC, containing green fluorescence ZsGreen-labeled degron ODC (Gdeg), was kindly provided by Dr. Frank Pajonk (UCLA’s Jonsson Comprehensive Cancer Center, CA, USA). The vector was transfected into Platinum retroviral packaging cells [25], and the retrovirus collected from the supernatant was used to infect pancreatic cancer cells. Stable transfectants were selected with G418 (Geneticin; PROMEGA, Madison, WI, USA), and the accumulation of ZsGreen-degronODC protein (Gdeg) was monitored by fluorescence microscopy and flow cytometry (FITC channel). To verify stable transfection, the cells were exposed to the proteasome inhibitor MG-132 (Calbiochem, San Diego, CA, USA) for 12 hours. Fluorescence microscopy was performed using AxioObserver (Carl Zeiss, Oberkochen, Germany), and the images were acquired digitally using AxioVision software (Carl Zeiss).

### In vitro cell migration and invasion assays

The double-chamber migration assay was performed by using a transwell chamber [26] (24-well plate, 8-μm pores; BD Biosciences, Canaan, CT, USA). For the invasion assay, matrigel-coated (BD Biosciences) transwells (0.1 mg/mL) were prepared by incubation in serum-free medium for 2 hours at 37°C in a 24-well plate. The lower chamber was filled with 0.8 mL culture medium without antibiotics. Then, DCLK1 high expression, wild-type, Gdeg^high^, and Gdeg^high^-siDCLK1 tumor cells (8 × 10^4^ in 0.3 mL serum-free medium) were seeded into the upper chamber and incubated at 37°C for 24 hours. The tumor cells on the upper surface of the filter were removed by using a cotton wool swab. Cells were then fixed with 100% methanol and stained with Giemsa solution, and the number of cells migrating or invading into the lower surface was counted in three randomly selected high-magnification fields (100×) for each sample. Each experiment was conducted in triplicate.

### Microarray analysis

For the gene expression analysis, 2 × 10^5^ freshly sorted Gdeg^high^ and Gdeg^low^ KLM1 cells were used. Total RNA was extracted from each cell type with QIAZOL, an RNeasy Micro Kit, and QIAshredder spin columns (QIAGEN, Hilden, Germany). The integrity of the RNA obtained was assessed with a NanoDrop ND-100 spectrophotometer (Thermo Fisher Scientific, Carlsbad, CA, USA), a 2100Bioanalyzer, and an RNA6000Pico Kit (Agilent Technologies, Palo Alto, CA, USA). According to the GeneChip®3’IVT Express Kit, P/N702646 Rev.7 protocol (Affymetrix, Santa Clara, CA, USA), complementary DNA was prepared from 100 ng total RNA by using one-cycle target labeling and a control reagent kit (Affymetrix). Hybridization and signal detection of HG-U133 Plus 2.0 arrays (Affymetrix) were performed. The microarray datasets of Gdeg^high^ and Gdeg^low^ cells were normalized and annotated with Expression Console™ Software (Affymetrix). Estimated gene expression levels were obtained as log2-transformed values. Genes were excluded from analysis if the detection call was “marginal” or “absent” in both Gdeg^high^ and Gdeg^low^ cells.

### Silencing and overexpression of *DCLK1*

Small interfering RNA (siRNA) targeting the candidate genes and scrambled siRNAs (siScr) not matching any human genes were purchased from Invitrogen. Freshly sorted Gdeg^high^ cells (both KLM1 and BxPC3) were seeded at a density of 2.0 × 10^5^ cells per well in 6-well plates in 2 mL culture medium. Thereafter, transfection with siRNA was performed by using RNAiMAX (Invitrogen, Carlsbad, CA, USA) according to the manufacturer’s instructions [27]. After transfection with various concentrations (10 nM, 20 nM), the cells were incubated for 48 hours at 37°C in a 5% CO_2_ atmosphere. The number of viable and nonviable cells was counted by an automatic cell counting machine by using trypan blue dye exclusion (CYTORECON; HIRATA, Tokyo, Japan). At the 48-hour time point after siRNA transfection, cells were detached from each plate to use for further experiments. The siRNA target sequences were those from high-content genome-wide RNAi screens [28]. To construct the shRNA expression vector (pSilencer^TM^ 2.1-U6 puro; Invitrogen) [29], KLM1-Gdeg cells (2.0 × 10^5^) were transfected with 2.5 μg plasmid with Lipofectamine2000 (Invitrogen). The medium was changed every 2 days, and stable transfectants were isolated by incubating in medium containing 400 μg/mL puromycin. To create transient tumor cells overexpressing DCLK1, we used pCMV6-AC-GFP (RG217050 TrueORF^TM^ cDNA Clones and PrecisionShuttle^TM^ Vector System, OriGene Technologies, Rockville, MD, USA). KLM1 cells were transfected using Lipofectamine2000 according to the manufacturer’s protocol. After incubation at 37°C for 48 hours, GFP-positive cells were sorted for further experiments.

### In vivo studies of tumorigenicity and metastasis

Female NOD.CB17-PRkdcScid/J mice, aged 5–6 weeks, were purchased from Charles River Laboratory (Kanagawa, Japan). Surgery was performed under anesthesia provided by isoflurane nose-cone inhalation. Various amounts of sorted cells (1 × 10^2^ to 1 × 10^5^ Gdeg^high^-KLM1 and Gdeg^low^-KLM1; 1 × 10^2^ to 1 × 10^4^ Gdeg^high^-BxPC3 and Gdeg^low^-BxPC3) were mixed with matrigel, and 100 μL was injected subcutaneously into both flanks of the mice. Tumor formation then was monitored every 4 days for up to 10 weeks. The tumor volume was estimated by using the following equation: volume = (length) × (width) ^2^ /2. Each group consisted of 4–6 mice. For liver metastasis analysis, the abdominal skin and muscle, approximately 5 mm in length, were incised just off the midline. The spleen was exteriorized through the incision by applying gentle traction, and the sorted cells (Gdeg^high^-KLM1, Gdeg^low^-KLM1 and Gdeg^high^-KLM1-shDCLK1, Gdeg^high^-BxPC3 and Gdeg^low^-BxPC3; n = 6), suspended at 1 × 10^6^ cells per 100 μL PBS, were injected just under the capsule of the lower pole by using a tuberculin syringe with a 30-gauge needle. Hemostasis was obtained by applying gentle pressure (with a cotton wool swab) on the injection site. The spleen was then returned to the peritoneal cavity, and the abdomen was closed by using two 6–0 silk sutures through both skin and muscle simultaneously [30, 31]. We observed these mice for 8 weeks, and then investigated whether metastasis had occurred [32]. All in vivo procedures in this study were approved by the Animal Care Committee of Tokyo Medical and Dental University (permission No.0150044A).

### Patients and samples

We enrolled patients who underwent primary and metastatic (liver and lung) tumor resection for pancreatic ductal adenocarcinoma at the Tokyo Medical and Dental University Hospital between 2005 and 2013. Paired samples were used for immunohistochemical analyses. Resected tissue was fixed in 10% formaldehyde solution and embedded in paraffin for histopathological analysis according to The General Rules for the Clinical and Pathological Study of Primary Pancreatic Cancer. This study was approved by ethics committee of Tokyo Medical and Dental University (Permission No.1080) and written informed consent was obtained from all patients.

### Statistical analysis

All values are presented as the mean ± SD unless otherwise stated. The two-tailed Student’s t-test (for comparison between groups) or one-way analysis of variance (for comparison of three or more groups) followed by Dunnett *post-hoc* tests were used for statistical analyses. SPSS software version 21.0 (SPSS, Chicago, IL) was used. Statistical significance was defined as p <0.05.

## Results

### Visualized pancreatic CSCs are particularly capable of liver metastasis

**Fig 1:**
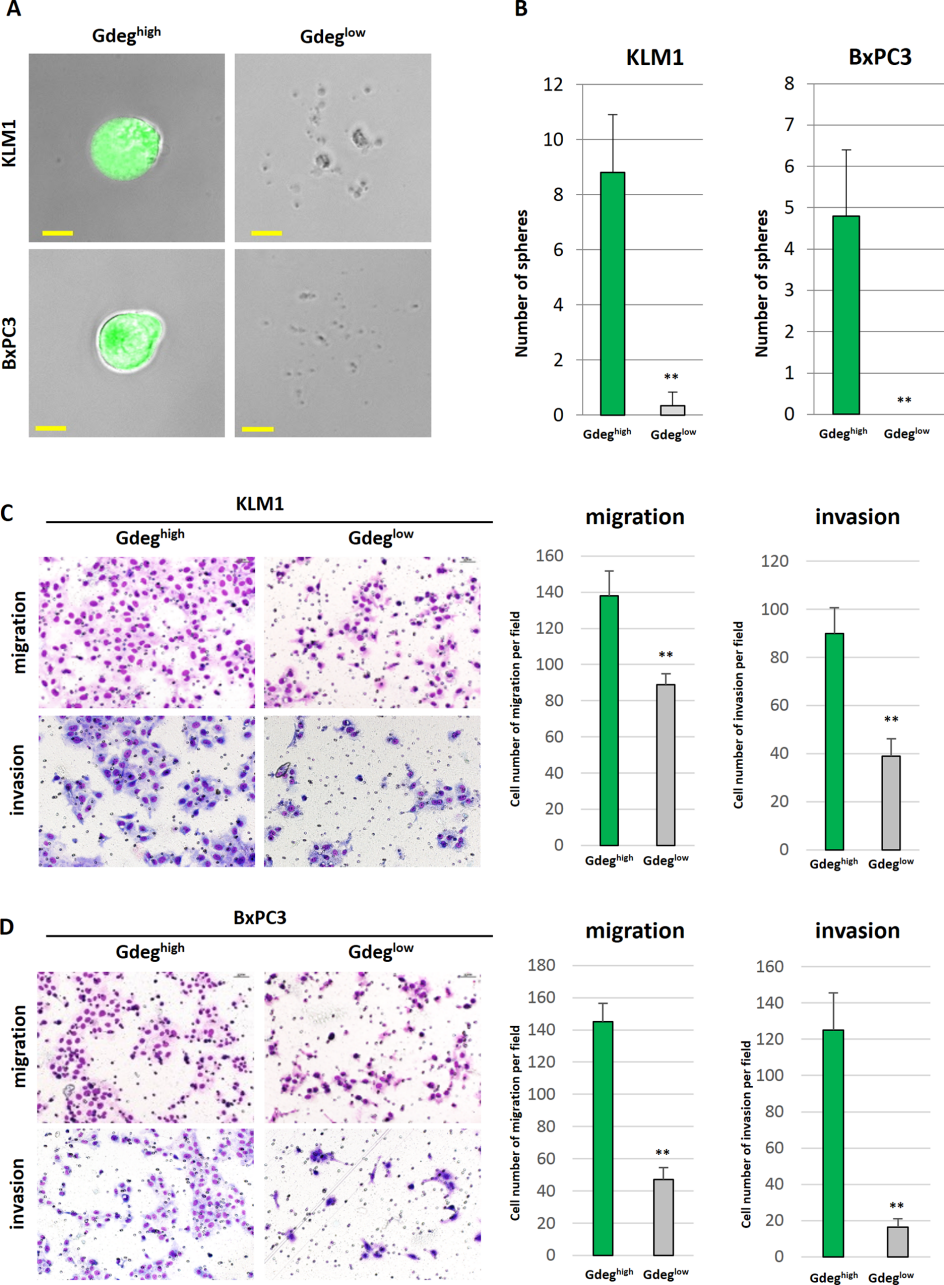
Sphere formation, migration, and invasion assay in Gdeg cell lines. (A) Representative microscopic images of spheres are shown. Gdeg^high^-KLM1 and Gdeg^high^-BxPC3 cells formed complete spheres, but Gdeg^low^-KLM1 and Gdeg^low^-BxPC3 cells did not. Scale bar, 50 μm. (B) The number of spheres (>50 μm in diameter) observed in each well (n = 6). Data are the mean ± SD. **p <0.01 compared with Gdeg^high^. The two-sided Student’s t-test was used to compare two independent groups. (C and D) Cell migration and invasion were analyzed with a double-chamber assay using Gdeg^high^-KLM1, Gdeg^low^-KLM1, Gdeg^high^-BxPC3, and Gdeg^low^-BxPC3 cells. Representative microscopic images are shown. Quantification of migration and invasion is presented as the mean ± SD for three independent experiments. **p <0.01 compared with Gdeg^high^ cells.

**Fig 2:**
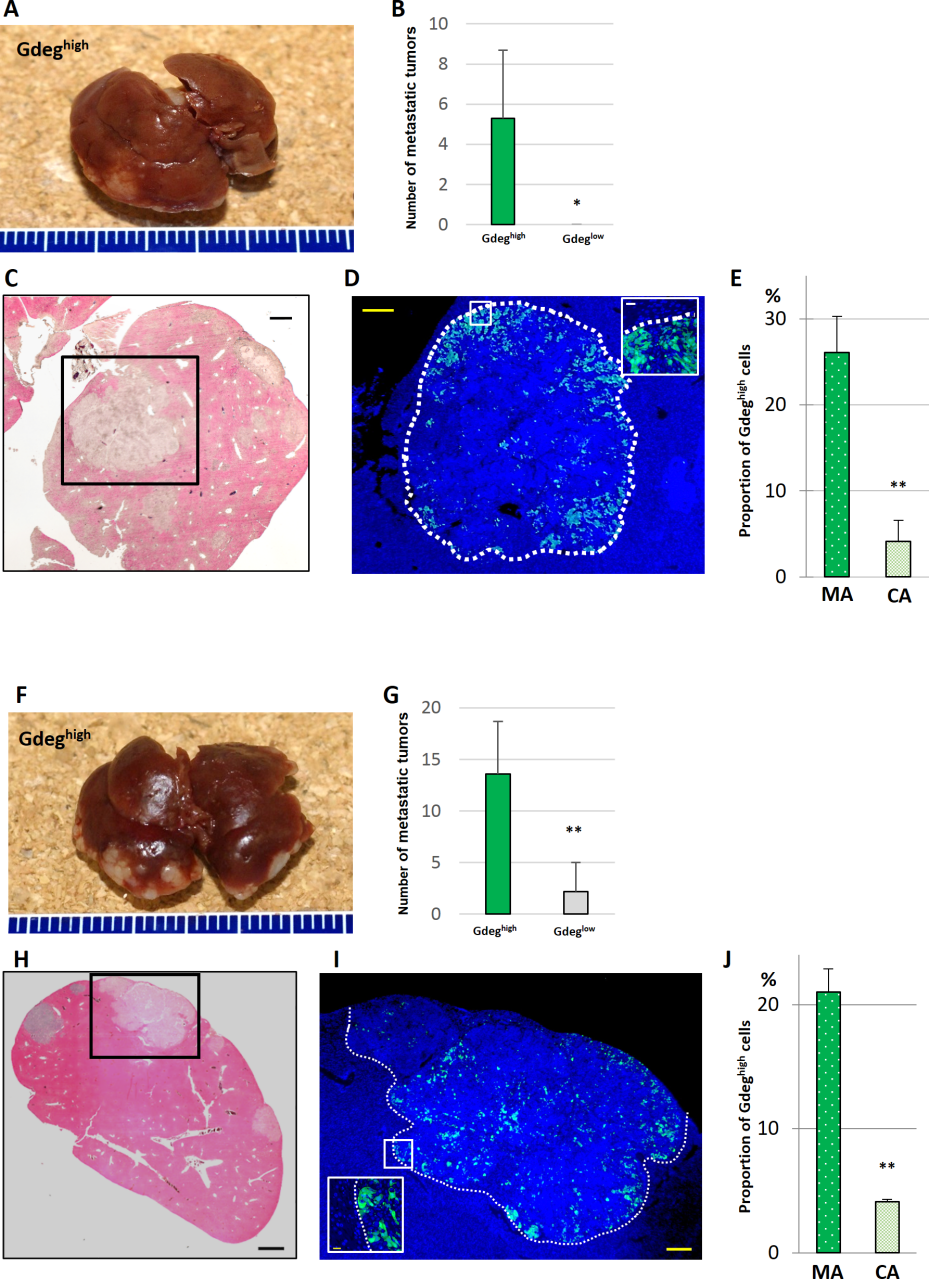
Liver metastatic model of Gdeg^high^-KLM1 and Gdeg^high^-BxPC3 cells in mice. (A and F) Representative liver metastatic tumors are shown in mice 8 weeks after injection. (B and G) Histogram shows the average number of tumors in the liver (n = 6; mean ± SD). For both cell lines, we found a significant difference between the Gdeg^high^ and Gdeg^low^ cells. *p <0.05 in (B) and **p <0.01 in (G). (C and H) Microscopic images of livers that were resected, sectioned, and stained with H&E are shown. Some micro-tumors were present in the liver. Scale bar, 1000 μm. (D and I) Fluorescent microscopic images of the main tumor are shown. For both cell lines, Gdeg^high^ cells were localized preferentially at the invading tumor margins. Scale bar, 200 μm. Magnified images of the boxed regions are shown. Scale bar, 20 μm. (E and J) The graph shows that the proportion of Gdeg^high^ cells was significantly higher in the marginal area (MA) than in the central area (CA; mean ± SD). **p <0.01. We counted three different areas for each region.

Stable transfection of the Gdeg reporter into two human pancreatic cancer cell lines with different oncogenic potentials of metastasis; KLM1 as high-ability cells with mutant KRAS, and BxPC3 as low-ability cells with wild-type KRAS [33–35]. CSCs labeled by reporter demonstrated a Gdeg^high^ population that accounted for approximately 1.0% of the total cell number (S1 Fig). In the assay for sphere formation [36], the number of spheres (>50 μm in diameter) derived from Gdeg^high^ cells was significantly higher than that of Gdeg^low^ cells (p <0.01, Fig 1A and 1B). Increased tumorigenicity in vivo has also been used as a piece of critical evidence for the existence of CSCs [4]. Thus, a sorted population of Gdeg^high^ or Gdeg^low^ cells was injected subcutaneously into NOD/SCID mice. For Gdeg^high^ cells derived from both the KLM1 and BxPC3 cell lines, we confirmed the highly malignant potential (S2 Fig). In both the KLM1 and BxPC3 cell lines, Gdeg^high^ cells had a significantly greater ability to migrate and invade than Gdeg^low^ cells in a double-chamber assay (p <0.01, Fig 1C and 1D). To investigate whether the Gdeg cells mediate metastasis in vivo, Gdeg^high^ or Gdeg^low^ cells were injected into the spleens of NOD/SCID mice (n = 6). After 8 weeks, we visually confirmed the presence of tumors in the spleen and counted the number of liver metastases. We found significantly more liver metastatic tumors in the Gdeg^high^ group than in the Gdeg^low^ group (p <0.05; Gdeg^high^-KLM1 cells, 5.3 ± 3.4 tumors versus Gdeg^low^-KLM1 cells, 0 ± 0 tumors, p <0.01; Gdeg^high^-BxPC3 cells, 13.6 ± 5.1 tumors versus Gdeg^low^-BxPC3 cells, 2.2 ± 2.8 tumors) (Fig 2A and 2B, 2F and 2G). We also observed some micro-metastases in the liver of Gdeg^high^ groups (Fig 2C and 2H), but we note that no metastatic tumors were seen in mice injected with Gdeg^low^-KLM1 cells. Immunofluorescent analysis clearly revealed Gdeg^high^ cells that were localized to the margins of the tumor (Fig 2D and 2I). The proportion of Gdeg^high^ cells in the tumor margin area (MA; 200 μm) was much higher than that in the tumor central area (CA) (Gdeg^high^-KLM1 tumor; 26.1 ± 4.2% versus 4.1 ± 2.5%, Gdeg^high^-BxPC3 tumor, 21.0 ± 1.9% versus 4.1 ± 0.2%, p <0.01) (Fig 2E and 2J). As in a previous study [11], our results suggested that the CSCs tended to invade further outside the tumor.

### Overexpression of DCLK1 in association with histone modification is responsible for the invasive potential of pancreatic CSCs

**Fig 3:**
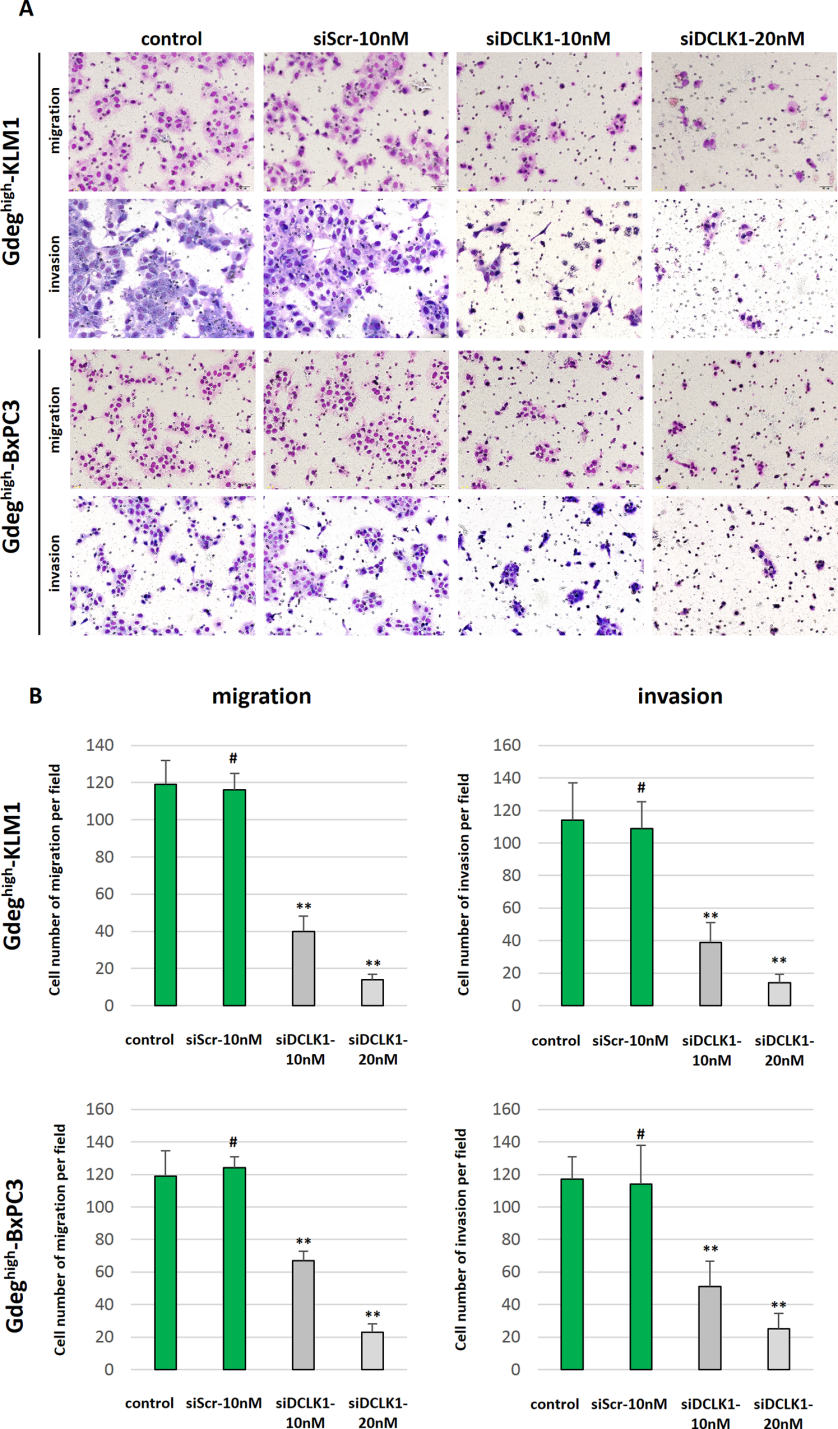
Knockdown of DCLK1 in Gdeg^high^-KLM1 and Gdeg^high^-BxPC3 cells suppresses cell migration and invasion in vitro. (A) Double-chamber assay of migration and invasion for both Gdeg^high^ cell lines after treatment. Representative microscopic images are shown. (B) The number of migrating and invading cells compared to the control was strikingly suppressed in both Gdeg^high^-siDCLK1 cell lines. #not significant, **p <0.01, by one-way analysis of variance followed by Dunnett multiple comparison tests.

**Fig 4:**
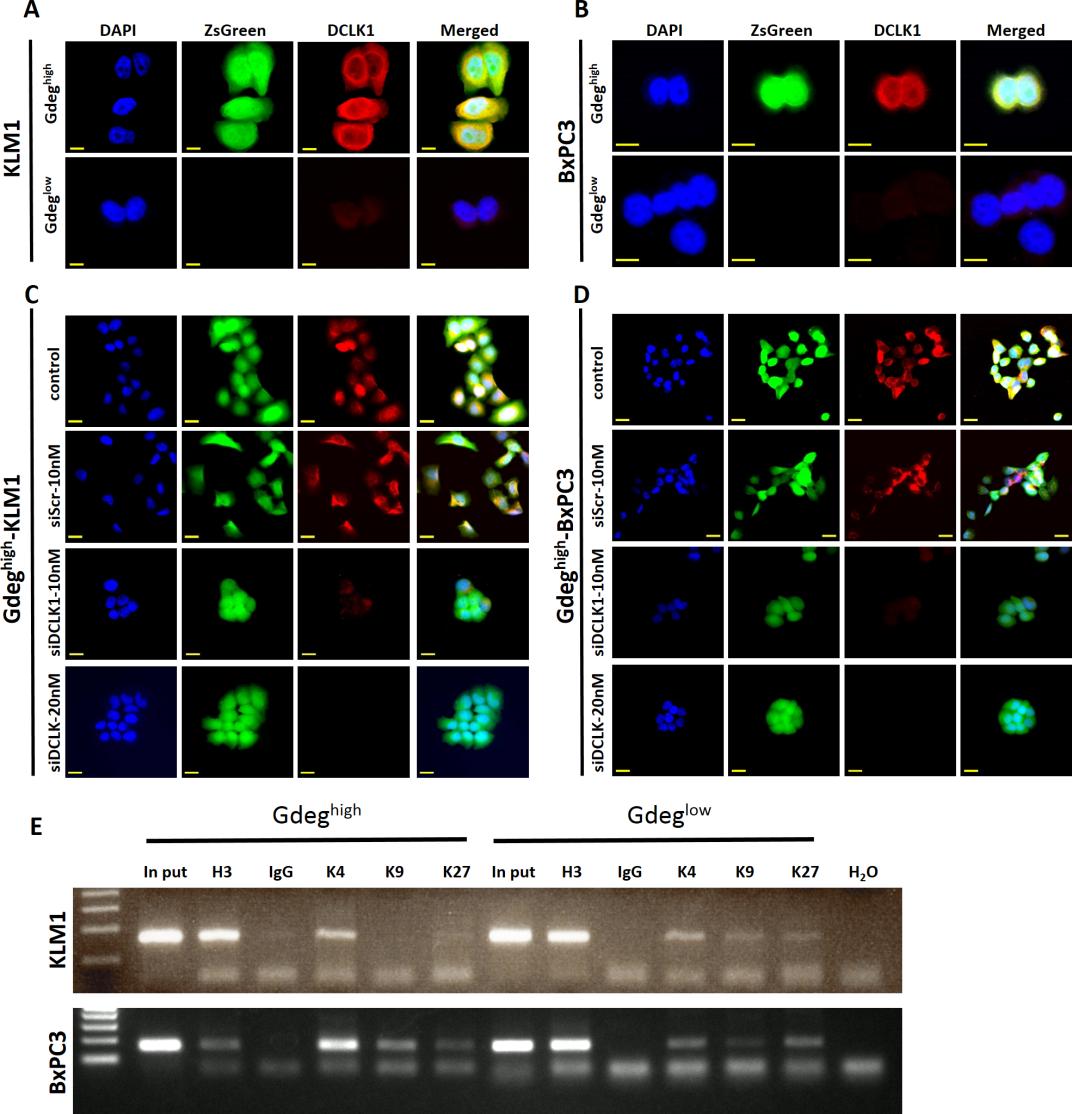
Immunocytochemical analysis of DCLK1 and ChIP analysis. (A and B) Immunocytochemical analyses of DCLK1 in both cell lines (original magnification ×200) are shown. Gdeg^high^ cells expressed DCLK1 protein, which was localized in the cytoplasm, but Gdeg^low^ cells showed only weak expression. Scale bar, 10 μm. (C and D) Immunocytochemical analysis of cells treated with *DCLK1*-specific siRNA is shown (original magnification ×200). DCLK1 protein expression was decreased in Gdeg^high^ cells after transfection with siDCLK1. Scale bar, 20 μm. (E) ChIP analysis is shown. Both Gdeg^high^ cell lines were strongly enriched for H3K4me3 compared to H3K27me3 at the *DCLK1* promoter region. Histone H3 and IgG were used as positive and negative controls for ChIP analysis, respectively.

To clarify the invasive and metastatic markers of pancreatic CSCs, we compared the gene expression patterns between Gdeg^high^ cells and Gdeg^low^ cells by microarray hybridization (S1 Data). Scatterplot analysis of the 25,572 probes for which the detection call was “present” showed that 483 genes were upregulated and 1,138 genes were downregulated by more than 2-fold in Gdeg^high^-KLM1 cells compared with Gdeg^low^-KLM1 cells. Based on the fold change ranking in the Gdeg^high^ cells (Table 1), siRNA screening for candidate genes was assessed according to the inhibitory effects on Gdeg^high^ cell migration and invasion using the double-chamber assay. Consequently, only the siRNA against DCLK1 significantly decreased cellular migration and invasion compared to controls in both KLM1 and BxPC3 Gdeg^high^ cells (p <0.01, Fig 3A and 3B). DCLK1 was identified as a target molecule that is overexpressed in Gdeg^high^ cells with migration and invasive ability compared to Gdeg^low^ cells. QRT-PCR and Western blotting analyses revealed that DCLK1 mRNA and protein was overexpressed in Gdeg^high^ cells but not in Gdeg^low^ cells (S3 Fig). Immunocytochemical analysis showed DCLK1 protein was mainly localized in the cytoplasm (Fig 4A and 4B), and reduced expression of DCLK1 was confirmed by the use of siRNA (S3 Fig, Fig 4C and 4D). Next, we examined epigenetic aspects of *DCLK1* expression by conducting chromatin immunoprecipitation (ChIP) analysis of three key histone methylation markers, H3K4me3, H3k9me3, and H3K27me3, in both Gdeg cell lines. As shown in Fig 4E, Gdeg^high^ cells expressed higher levels of H3K4me3, which is associated with active promoters, than H3K27me3, which is associated with silenced promoters. Gdeg^low^ cells were nearly bivalent. These results imply that *DCLK1* expression in Gdeg cell lines was regulated by histone modification of H3K4 and H3K27. We found no difference in H3K9me3 levels between Gdeg^high^ and Gdeg^low^ in these cell lines.

**Table 1:** Differentially expressed genes in Gdeg^high^ -KLM1 cells ranked by fold change (FC).

Rank	Probe set ID	Gene symbol	Signal		FC(log2)	siRNA target sequence
			Gdeg^high^	Gdeg^low^		
**1**	**214580_x_at**	**KRT6A/B/C**	**2882.3**	**94.0**	**4.938**	**- - - - -**
**2**	**209125_at**	**KRT6A**	**6639.6**	**303.8**	**4.450**	**5’ -GAGCUUCACUGUUACUAAA- 3’**
**3**	**205399_at**	**DCLK1**	**4054.2**	**344.2**	**3.558**	**5’ -GACUUUGGACUGGCCACCA- 3’**
**4**	**205767_at**	**EREG**	**1402.7**	**157.9**	**3.151**	**5’ -CCACCAACCUUUAAGCAAA- 3’**
**5**	**205064_at**	**SPRR1B**	**2203.3**	**260.3**	**3.081**	**5’ -AAAUGAUUCAGCUCCCUUA- 3’**
**6**	**215303_at**	**DCLK1**	**370.2**	**46.0**	**3.010**	**(5’ -GACUUUGGACUGGCCACCA- 3’)**
**7**	**230962_at**	**DCLK1**	**280.4**	**36.5**	**2.942**	**(5’ -GACUUUGGACUGGCCACCA- 3’)**
**8**	**209126_x_at**	**KRT6B**	**2385.9**	**325.6**	**2.873**	**5’ -CAACAGUUAUCAGCACUCA- 3’**
**9**	**241268_x_at**	**- - - - -**	**172.9**	**24.2**	**2.836**	**- - - - -**
**10**	**204472_at**	**GEM**	**952.7**	**136.2**	**2.806**	**- - - - -**
**11**	**214321_at**	**NOV**	**473.8**	**79.7**	**2.572**	**5’ -CAGGGCAAAUAGUCAAGAA- 3’**
**12**	**1556773_at**	**- - - - -**	**732.6**	**136.0**	**2.429**	**- - - - -**
**13**	**207173_x_at**	**CDH11**	**231.6**	**45.6**	**2.345**	**5’ -GAAUCCUGAUGGUAUCAAU- 3’**
**14**	**209351_at**	**KRT14**	**1944.1**	**382.8**	**2.345**	**5’ -GGUCAUGGAUGUGCACGAU- 3’**
**15**	**224480_s_at**	**AGPAT9**	**5905.5**	**1190.3**	**2.311**	**5’ -CCAGUUGCAAUUAAGUAUA- 3’**
**16**	**202436_s_at**	**CYP1B1**	**3803.6**	**767.4**	**2.309**	**5’ -CAGUUAUGGUCUAACCAUU- 3’**
**17**	**224941_a**	**PAPPA**	**175.2**	**35.7**	**2.293**	**5’ -GAGCCUACUUGGAUGUUAA- 3’**
**18**	**202437_s_at**	**CYP1B1**	**3584.7**	**744.7**	**2.267**	**(5’ -CAGUUAUGGUCUAACCAUU- 3’)**
**19**	**202435_s_at**	**CYP1B1**	**2693.5**	**564.4**	**2.255**	**(5’ -CAGUUAUGGUCUAACCAUU- 3’)**
**20**	**235608_at**	**- - - - -**	**235.9**	**49.6**	**2.248**	**- - - - -**

### DCLK1 promotes morphological changes, migration, and invasion in human pancreatic cancer cells

**Fig 5:**
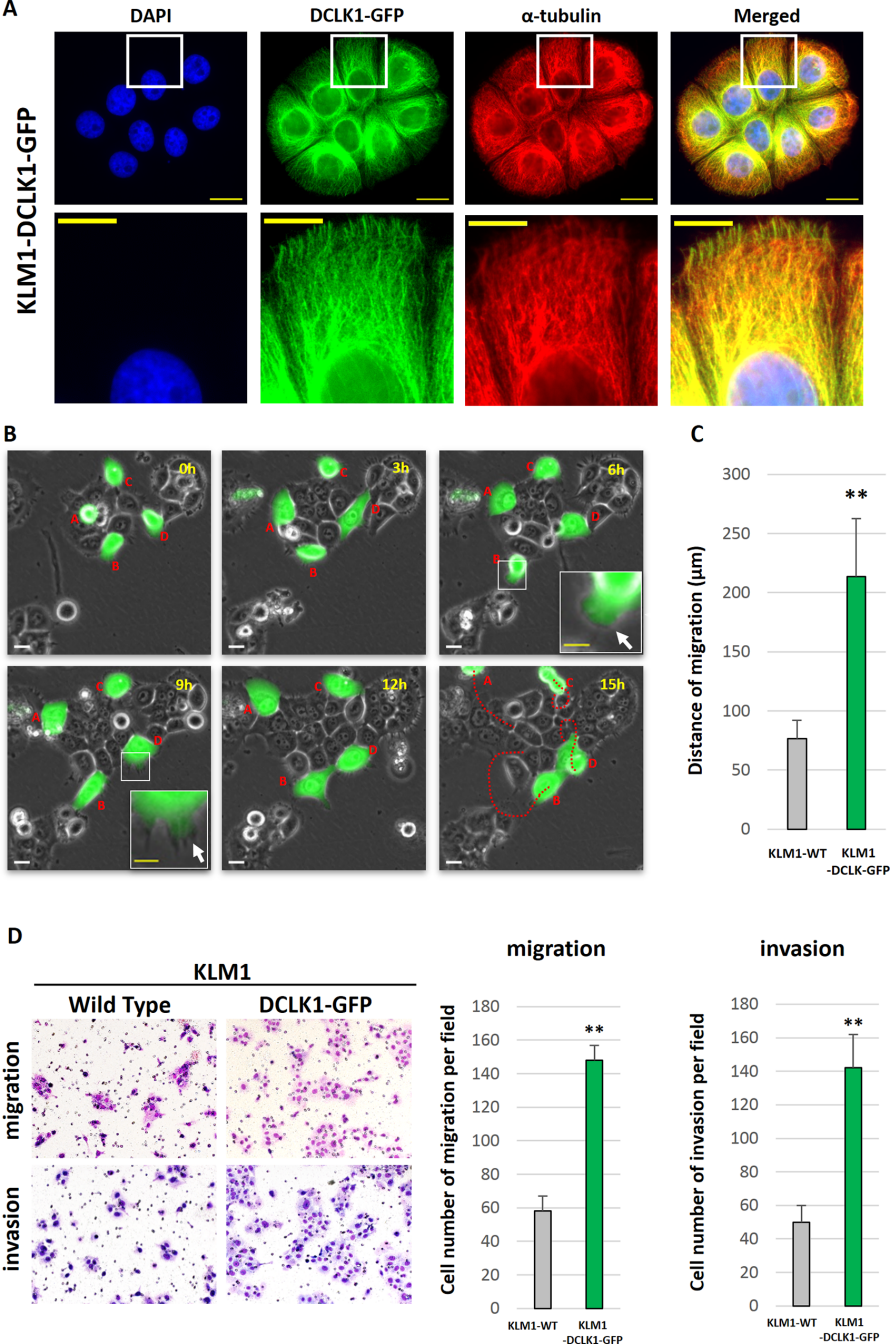
Gain-of-function analysis shows the effects of DCLK1 overexpression in KLM1 (KLM1-DCLK1-GFP) cells in vitro. (A) Immunocytochemical analysis showed that DCLK1 was predominantly co-localized with α-tubulin in KLM1-DCLK1-GFP cells. Scale bar, 20 μm. Magnified images of the boxed regions are shown in the second row. Scale bar, 10 μm. (B) Four KLM1-DCLK1-GFP cells were observed with time-lapse photography for 15 hours. They adopted an amoeboid morphology as they moved. Scale bar, 20 μm. A magnified image of the boxed regions is shown in the corner. White arrows mark stretching pseudopodia. Scale bar, 10 μm. Their tracks (shown as red dotted lines) were measured. (C) Histogram shows the average distance of migration for wild-type KLM1 cells (control cells) and KLM1-DCLK1-GFP cells (n = 4 each, mean ± SD). KLM1-DCLK1-GFP cells migrated significantly longer than control cells. **p <0.01. (D) The number of migrating and invading KLM1-DCLK1-GFP cells was also higher than that of control cells. **p <0.01.

To investigate the role of DCLK1 in human pancreatic cancer cells, we adopted a gain-of-function strategy by using KLM1 cells that transiently over-expressed GFP-tagged DCLK1. As previously reported for neuronal cell lines, immunofluorescence analysis of KLM1-DCLK1-GFP demonstrated overlapping patterns of DCLK1 expression with microtubules by using an α-tubulin antibody [37] (Fig 5A). The function of DCLK1 in human pancreatic cancer cells may thus be to control the action of microtubules. Movement and morphological changes including stretching pseudopodia were clearly observed in time-lapse microscopic images (Fig 5B). During a 15-hour observation period, the migration distance of KLM1-DCLK1-GFP cells was longer than that of wild-type KLM1 cells (p <0.01, Fig 5C). In the double-chamber assay, the number of migrating and invading KLM1-DCLK1-GFP cells was higher than that of wild-type KLM1 cells (p <0.01, Fig 5D). Moreover, the KLM1-DCLK1-GFP cells trans-located via rapidly alternating cycles of morphological expansion and contraction called amoeboid migration (S1 Video) [38].

### DCLK1 knockdown leads to a reduction in the metastatic ability of pancreatic CSCs

**Fig 6:**
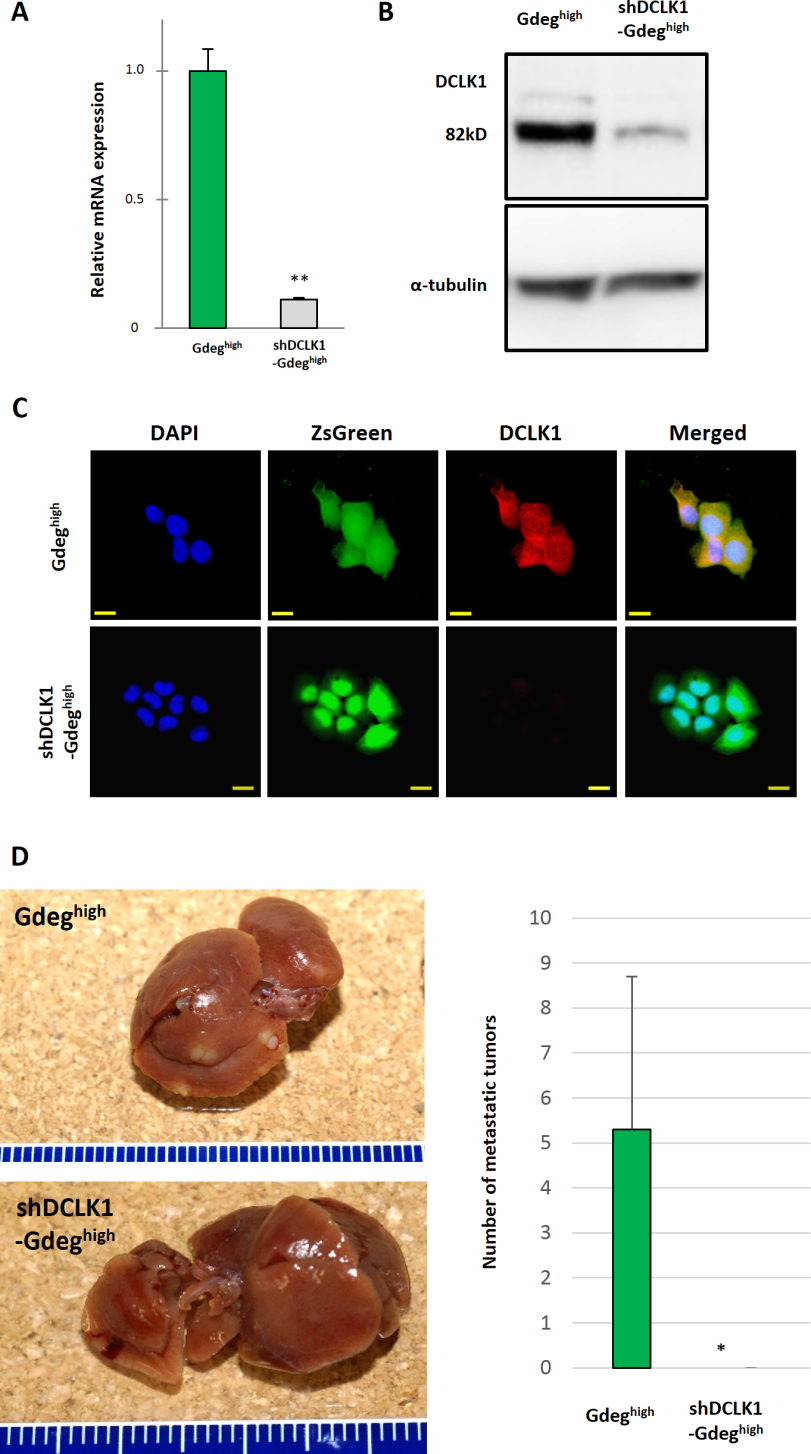
Knockdown of DCLK1 in Gdeg^high^-KLM1 cells shows DCLK1 is essential for tumor metastasis in vivo. (A and B) *DCLK1*-specific shRNA (shDCLK1) significantly decreased DCLK1 mRNA and protein expression in Gdeg^high^-KLM1 cells. **p <0.01. (C) A marked decrease in DCLK1 immunoreactivity was observed following transfection with shDCLK1 compared to Gdeg^high^ (control). Scale bar, 20 μm. (D) Some tumors along the edge of the liver were visible in the Gdeg^high^-KLM1 group, but no tumors were seen in the shDCLK1-Gdeg^high^ group. The average number of tumors in the liver (n = 6 mice) is shown (mean ± SD). We observed a significant difference between the control group and the shDCLK1 group. *p <0.05.

To confirm the in vivo loss-of-function analysis, stable knockdown of DCLK1 was assessed in Gdeg-KLM1 cells using *DCLK1* shRNA. The quality of transfection was confirmed by qRT-PCR, western blotting and immunocytochemical analyses (Fig 6A–6C). The population of shDCLK1-Gdeg^high^-KLM1 cells was less than 0.5% of the total cell number (S1 Fig). We injected freshly sorted shDCLK1-Gdeg^high^-KLM1 cells into the spleens of NOD/SCID mice (n = 6). Significant liver metastases were detected following injection of control Gdeg^high^ cells, but interestingly, no metastatic lesions were identified following injection of shDCLK1-Gdeg^high^ cells, as determined by macroscopic and microscopic examination (Fig 6D). These results implied that DCLK1 plays an essential role in liver metastasis in human pancreatic cancer.

### Dominant expression of DCLK1 in metastatic tumors is clinically recognized in patients with pancreatic cancer

**Fig 7:**
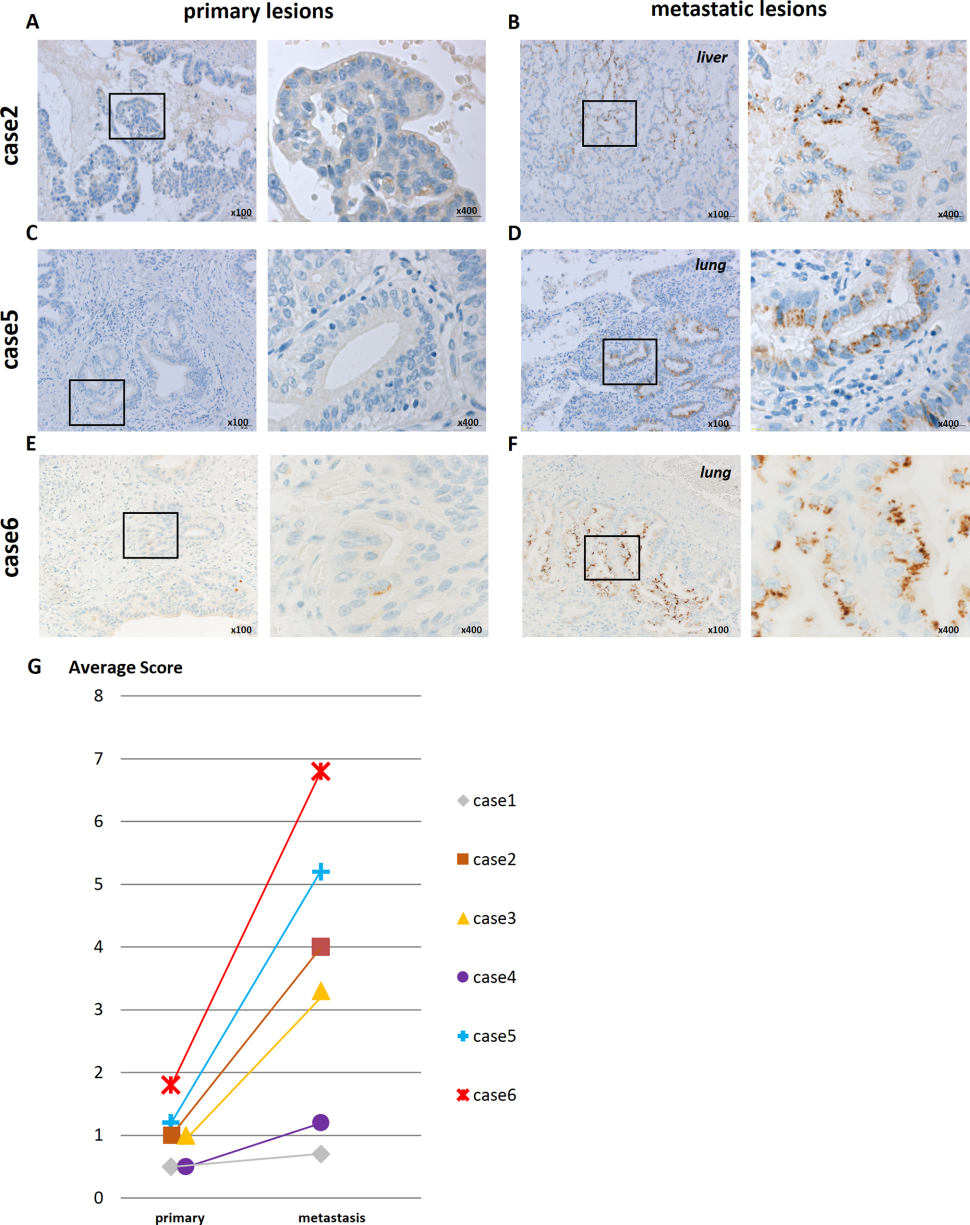
Immunohistochemical analysis of DCLK1 expression in clinical samples. (A, C, and E) In primary lesions, few cancer cells were positive for DCLK1, and the staining intensity was weak (left; 100×, right; 400×). (B, D, and F) In metastatic lesions (liver or lung), the number of DCLK1-expressing cells was higher than in primary tumors, and the staining intensity was stronger (left; 100×, right; 400×). (G) Comparison of immunostaining scores between primary lesions and metastatic lesions. In all six clinical samples, DCLK1 was more highly expressed in the metastatic lesion than in the primary lesion.

Among 135 pancreatic cancer patients who underwent surgical resection from 2005 to 2013 in our hospital, six metastatic tumors were resected synchronously or metachronously. These six pairs of primary and metastatic tumors were evaluated for the expression of DCLK1. Primary and metastatic tumors were resected synchronously in three cases, and primary tumors and metachronous distant metastases were separately resected in the other three cases (Table 2). Interestingly, cancer cells that were positively stained for DCLK1 protein were rarely detected in the primary tumors, but were clearly detected in metastatic tumors (Fig 7A–7F), and in all cases, the staining score was higher in metastatic tumors than in primary tumors (Fig 7G). In cases 5 and 6 in particular, the primary tumors were completely resected and no local recurrence occurred. However, even though these patients were treated with adjuvant chemotherapy, distant metastasis appeared. In these two cases, DCLK1 expression was much higher in the metastatic tumor than in the primary tumor. These results suggest that metastatic tumors are resistant to conventional chemotherapy and that DCLK1 is a marker of metastatic tumors.

**Table 2:** Patient characteristics.

Case	Age/Gender	Synchronous/Metachronous	Metastatic Organ	Chemo(radio)therapy	Survival time after metastasis resection
1	67/M	Synchronous	Liver	TS-1/Gemcitabine	23 months
2	51/M	Synchronous	Liver	TS-1/Gemcitabine	6 months
3	77/M	Synchronous	Liver	TS-1/Gemcitabine/Erlotinib	11 months
4	59/M	Metachronous	Liver	TS-1/Gemcitabine	10 months
5	32/M	Metachronous	Lung	TS-1/Gemcitabine	24 months
6	60/F	Metachronous	Lung	TS-1/Gemcitabine/Radiation	12 months

## Discussion

DCLK1 consists of an N-terminus that is 65% similar to doublecortin (DCX) throughout the entire length of DCX, but DCLK1 also contains an additional 360 amino acids in the C-terminal domain, creating a putative Ca^2+^/calmodulin-dependent protein kinase. *DCLK1* is located on the region of chromosome 13q13.3 [37]. The *DCX* gene, which is located on chromosome region Xq23, is a microtubule-associated protein required for migration of neural progenitor cells to the cerebral cortex.

Mutations in DCX lead to cortical lamination defects in the developing brain that are called subcortical band heterotopia in females and classical lissencephalies in males [39]. A previous mouse study in which DCLK1 and/or DCX was knocked out revealed that both proteins have genetically compensatory roles in neuronal migration; that is, the *DCLK1* gene functions in a partially redundant pathway with DCX in the formation of axonal projections across the midline and migration of cortical neurons [40]. We found that overexpressed DCLK1 promotes migration and invasion in cell lines, and knockdown suppresses these abilities. We carefully considered how our results regarding DCLK1 fit with past results regarding a neuronal role for DCLK1. Some evidence exists for the potential involvement of axon guidance genes in pancreatic carcinogenesis [41], and these results are consistent with our finding that highly expressed DCLK1 is closely related to invasion and metastasis in pancreatic cancer. Epigenetic control of gene expression plays a major role in cell fate determination. In particular, higher-order chromatin structure is emerging as an important regulator of stem cell differentiation [8], including even pancreatic development [42]. During the induction of pluripotency, appropriate chromatin remodeling is required for expression of stem cell-specific genes. Furthermore, several studies on brain tumors, hepatocellular carcinoma and breast cancers have shown that histone modification is responsible for the hierarchical structure that comprises cancer stem cells at the apex [43–45]. Rheinbay *et al*. reported the loss of H3K27me3 activates a signaling pathway required for the maintenance and tumorigenicity of glioblastoma CSCs [43]. Knockdown of H3K20 methyltransferase PR-SET7 in livers induced spontaneous development of hepatocellular carcinoma with cancer stem cell properties [44]. Westcott *et al*. reported an epigenetically distinct subpopulation of breast tumor cells with an enhanced capacity to collectively invade [45]. In this study, ChIP analysis revealed that the *DCLK1* transcription start site in both Gdeg^high^ cell lines was more strongly marked with histone modification for gene activation (H3K4me3) than repression (H3K27me3). These data are valuable for showing the relationship between CSCs and epigenetic regulation [46].

Why do cells that express high levels of DCLK1 show enhanced migration, invasion, and metastasis in human pancreatic ductal adenocarcinoma? In our time-lapse experiments, we found that cells with high levels of DCLK1 exhibited amoeba-like changes in morphology. The process of tumor cell invasion and metastasis is conventionally understood as the migration of individual cells that detach from the primary tumor, enter lymphatic vessels or the bloodstream, and seed in distant organs. Mesenchymal and amoeboid types of cancer cells, which are different morphological variants of cancer cells, can undergo single-cell migration [38]. Thus, we believe that the amoeboid morphology may be the reason that Gdeg^high^ cells can invade and metastasize aggressively. Another study suggested that DCX is phosphorylated by Rho-kinase and that this phosphorylation is correlated with in vitro tumor cell migration, as well as in vivo invasion and progression [47]. However, additional details about the mechanism are unknown.

Accumulating evidence has indicated pancreatic CSCs are distinguished as highly invasive and metastatic abilities [48, 49]. Hermann *et al*. identified a distinct subpopulation of CD133+CXCR4+ CSCs in the invasive front of pancreatic tumors as the metastatic phenotype [11]. Our in vivo results revealed that Gdeg^high^ cells were located at the marginal region of the metastatic tumors, and knockout of DCLK1 significantly inhibited liver metastasis. We surveyed the expression of CXCR4 with qRT-PCR, but we found no difference between Gdeg^high^ and Gdeg^low^ cells (data not shown). Different mechanisms may regulate invasion and metastasis by CSCs, because these CSCs demonstrated a similar localization in the invading front of tumor margins (Fig 2D and 2I) and indispensable potential for in vivo metastasis (Fig 6D). Thus, the invasive and metastatic feature generally characterizes the pancreatic CSCs. Some studies implicated a relationship between *DCLK1* and microRNA, especially Let-7a or miR-200a [50, 51]. They reported that DCLK1 regulates epithelial-mesenchymal transition in human pancreatic cells through a miR-200a-dependent mechanism. However, our microarray data analysis (S1 Data) identified no difference between Gdeg^high^ and Gdeg^low^ cells regarding epithelial-mesenchymal transition-associated factors such as Twist, Slug, Snail, and Vimentin. Moreover, comprehensive microRNA analysis did not reveal a high expression level of miR-200a in Gdeg^high^ cells (data not shown).

DCLK1 has been thoroughly investigated in neuroscience and is vital for neuroblast proliferation, migration, and differentiation [52]. Carla *et al*. reported that the *DCLK1* gene is an attractive molecular target for neuroblastoma therapy [53]. DCLK1 plays a biological role in gastrointestinal tumors, and small molecule inhibitors of DCLK1 may be useful as anti-tumor stem cell drugs in these tumors [54–56]. Despite clinical trials of new therapies, no significant improvements in pancreatic cancer treatments have occurred. A new strategy, neoadjuvant chemotherapy, has been used to control local invasion and early micrometastasis before surgery. In the present study, we found high expression levels of DCLK1 in clinical samples from two different types of metastatic tumors (liver and lung). DCLK1 may be an essential factor in invasion and metastatic mechanisms and therefore could be a promising therapeutic target for treatment of pancreatic cancer.

## Supporting Information

S1 Data Gene expression patterns between Gdeg^high^ -KLM1 cells and Gdeg^low^ -KLM1 cells. (XLSX)

S1 Fig Representative images of the cultured cells after transfection. (TIF)

S2 Fig Enhanced tumor formation and tumorigenicity by Gdeg^high^ cells. (TIF)

S3 Fig Quantitative real-time RT-PCR analysis of mRNA expression and western blot analysis. (TIF)

S1 Methods Cell culture, Flow cytometry and cell sorting, Time-lapse analysis, Sphere formation assay, Quantitative real-time RT-PCR, ChIP and template preparation, Immunocytochemical and immunohistochemical analyses, Western blotting. (DOCX)

S1 Video Real time imaging of KLM1-DCLK1-GFP cells. (MP4)

## Abbreviations

CSC: cancer stem cell
ODC: ornithine decarboxylase
Gdeg: ZsGreen-labeled degron ODC
H3K4me3: histone H3 lysine 4 tri-methylation
H3K9me3: histone H3 lysine 9 tri-methylation
H3K27me3: histone H3 lysine 27 tri-methylation
FACS: fluorescence-activated cell sorting
RT-PCR: Reverse transcription-polymerase chain reaction
qRT-PCR: quantitative RT-PCR
GAPDH: glyceraldehyde-3-phosphate dehydrogenase
