# Supplementary figures and images for: Dominant Expression of DCLK1 in Human Pancreatic Cancer Stem Cells Accelerates Tumor Invasion and Metastasis
Source: PLoS One. 2016 Jan 14;11(1):e0146564. doi: 10.1371/journal.pone.0146564 (PMC4713149; doi:10.1371/journal.pone.0146564)

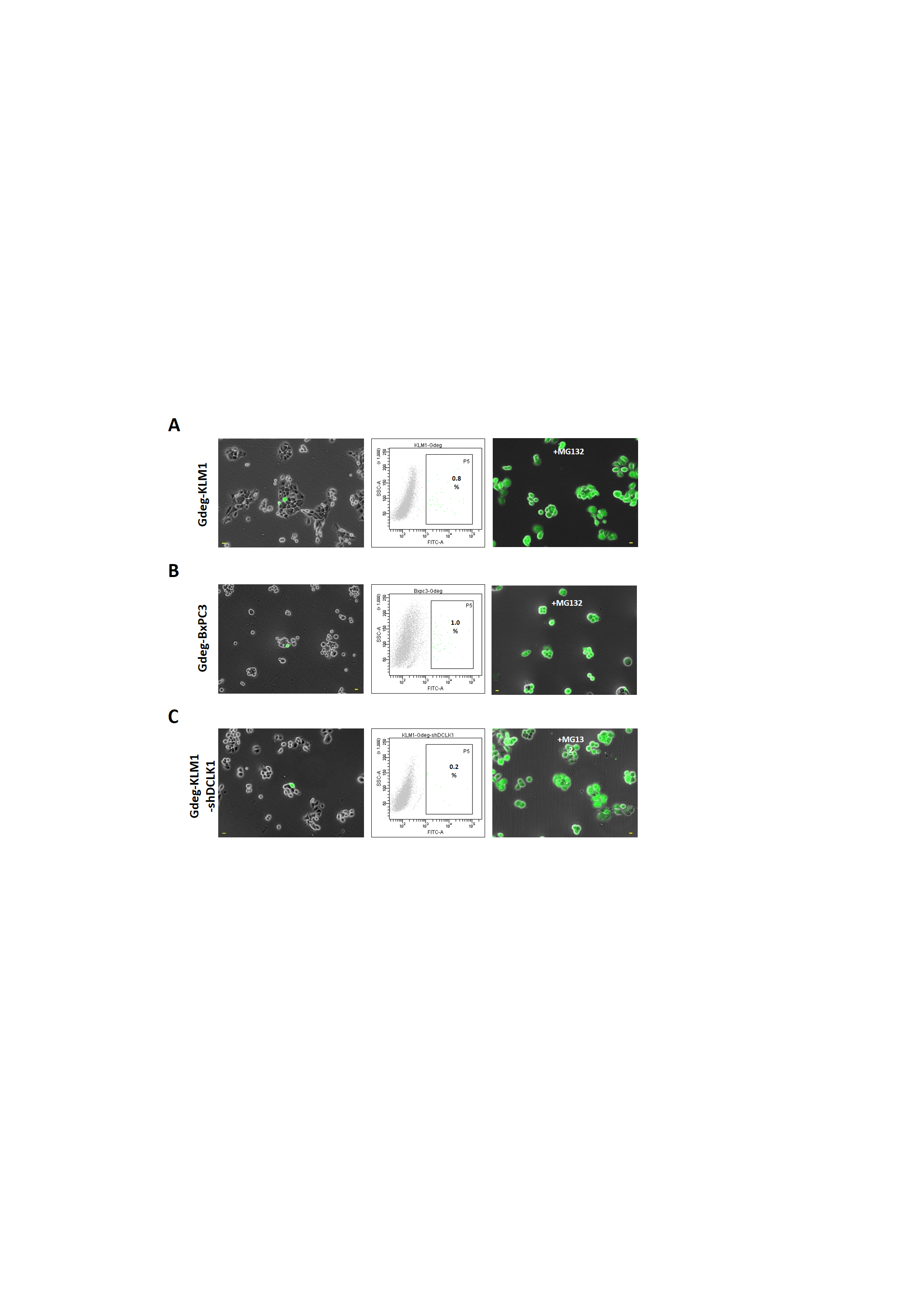

Supplement: S1 Fig — (TIF) [file pone.0146564.s002.tif]

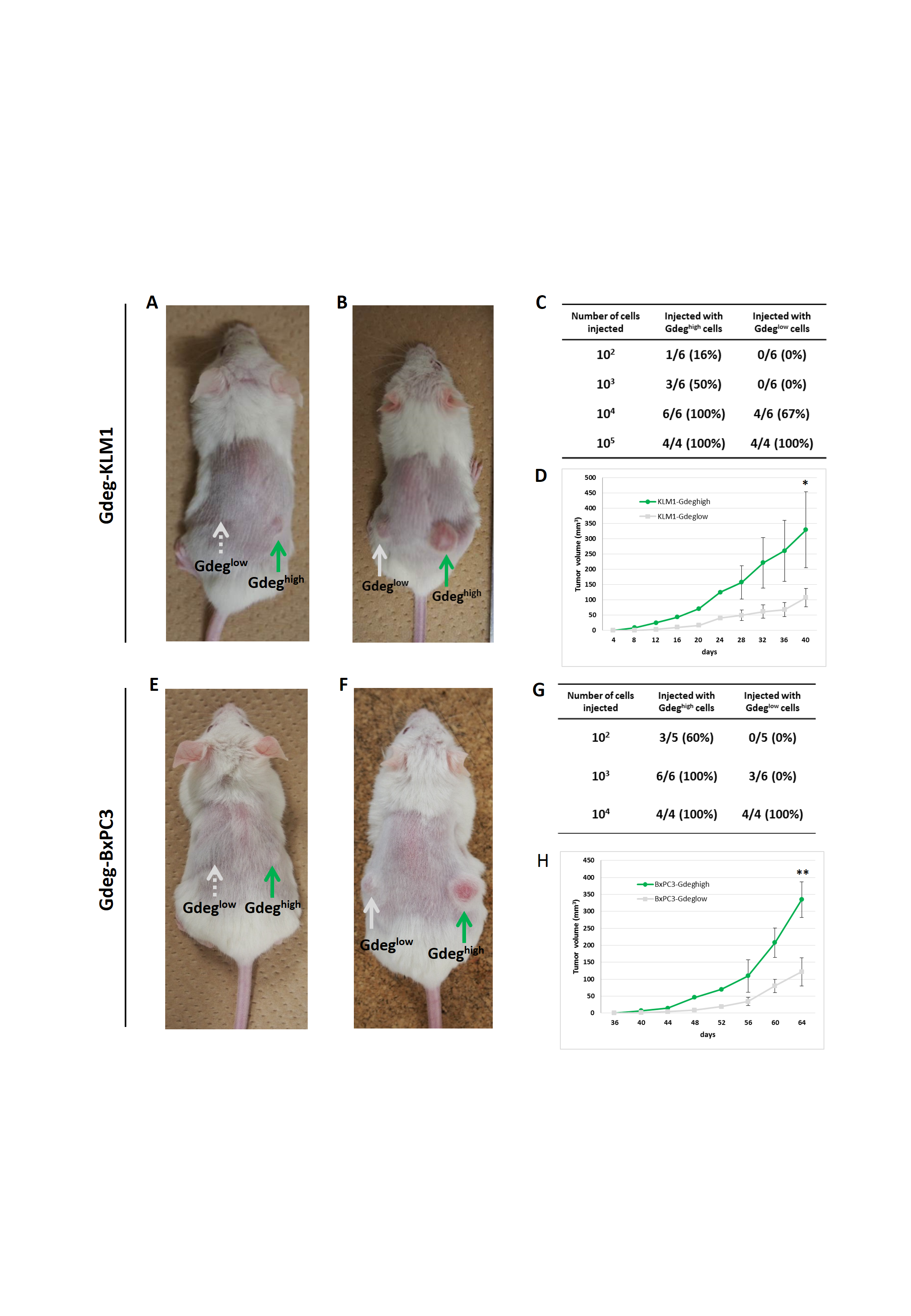

Supplement: S2 Fig — (TIF) [file pone.0146564.s003.tif]

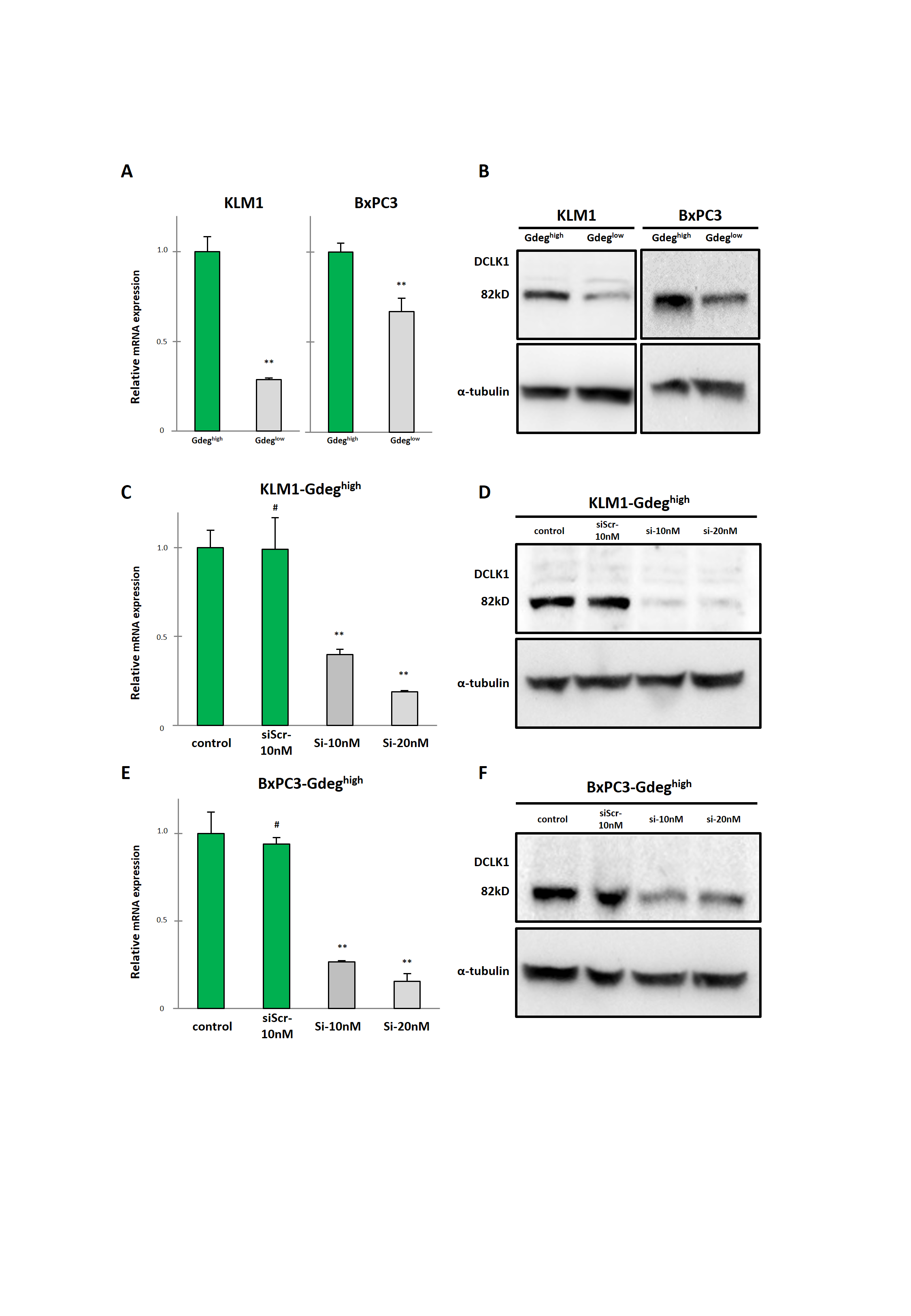

Supplement: S3 Fig — (TIF) [file pone.0146564.s004.tif]
