# Supplementary material for: Dominant Expression of DCLK1 in Human Pancreatic Cancer Stem Cells Accelerates Tumor Invasion and Metastasis
Source: PLoS One. 2016 Jan 14;11(1):e0146564. doi: 10.1371/journal.pone.0146564 (PMC4713149; doi:10.1371/journal.pone.0146564)
Supplement: S1 Methods — (DOCX) [file pone.0146564.s005.docx]

**S1 Methods File**

**Cell culture**

The human pancreatic adenocarcinoma cell line, BxPC3, was obtained from the American Type Culture Collection (Manassas, VA, USA). The other human pancreatic adenocarcinoma cell line, KLM-1, was newly purchased from RIKEN CELL BANK (Tsukuba, Japan). These two cell lines were grown in 1640 RPMI (Invitrogen, Carlsbad, CA) supplemented with 10% fetal bovine serum (Sigma, St. Louis, MO, USA) and 1% penicillin-streptomycin (Sigma) in a humidified chamber at 37 °C with 5% CO_2_.

**Flow cytometry and cell sorting**

For flow cytometry and cell sorting, we used FACSAria II (BD Bioscience). Pancreatic cancer cells were washed with phosphate-buffered saline and then enzymatically dissociated with 0.05% trypsin-EDTA (Invitrogen). Complete growth medium was added to the trypsinized cells, which were centrifuged at 1500 rpm for 5 minutes. We resuspended the cell pellet to a density of 1 × 10^6^ cells per mL for use. After sorting, cells were promptly dispensed into a culture flask.

**Time-lapse analysis**

Live cells were plated at a density of 1 × 10^5^ cells in a 6-cm dish containing culture medium. After incubation in 5% CO_2_ at 37 °C overnight, cell attachment was confirmed. Image analysis was performed by using AxioVision and AxioObserver (Carl Zeiss).

**Sphere formation assay**

After FACS sorting, Gdeg^high^ or Gdeg^low^ cells were plated separately at 1 × 10^2^ cells in low attachment plates (96-well Ultra Low Cluster Plate; Costar, Corning, NY, USA) and incubated in serum-free medium (n = 6 each). Sphere formation was observed after 48 hours using AxioObserver (Carl Zeiss), and the images were acquired digitally using AxioVision software (Carl Zeiss). Spheres were defined as clumps of cells with a diameter more than 50 μm.

**Quantitative real-time RT-PCR**

Total RNA was extracted from sorted cells by using an RNeasy Mini Kit according to the manufacturer’s instructions (QIAGEN). Quantitative real-time reverse transcription PCR was performed by using the Applied Biosystems®7500 Real-Time PCR system (Applied Biosystems, Foster City, CA, USA). Single-stranded cDNA was synthesized by using RT Master Mix. Quantitative PCR was performed by using the TaqMan® Gene Expression Master Mix and Assay. The assay IDs were: *DCLK1* Hs00178027_m1 and GAPDH Hs02786624_g1. The relative mRNA expression of *DCLK1* was calculated using the comparative threshold method with GAPDH for normalization.

**ChIP and template preparation**

Chromatin immunoprecipitation (ChIP) was performed using the ChIP-IT™ Express kit (Active Motif, Carlsbad, CA, USA) according to the manufacturer’s protocol. Sufficient freshly sorted Gdeg^high^ and Gdeg^low^ KLM1 and BxPC3 cells were prepared and then cross-linked with 1% formaldehyde for 10 minutes at room temperature. After sonication, ChIP analysis was conducted using antibodies for anti-tri-methylated histone H3 at lysine 4 (H3K4me3) (Cat. No. 39915, Active Motif), anti-tri-methylated histone H3 at lysine 9 (H3K9me3) (No. 39239, Active Motif), and anti-tri-methylated histone H3 at lysine 27 (H3K27me3) (No. 39155, Active Motif). Histone H3 (No. 39163, Active Motif) and normal rabbit IgG (No. 2729, Cell Signaling Technology, Danvers, MA, USA) were used as positive and negative controls for ChIP analysis, respectively. Input DNA samples were also used as an internal control. According to genomic *DCLK1* sequence data on the UCSC Genome Bioinformatics Site (http://genome.ucsc.edu/), we designed specific *DCLK1* primers and then performed PCR. The PCR products were electrophoresed in 2.5% agarose gels. The primer sequences of *DCLK1* and its PCR conditions are available upon request.

**Immunocytochemical and immunohistochemical analyses**

Cells were sorted with FACSAria II and then incubated for 24 hours on glass slides. After fixation with 4% formaldehyde for 20 minutes at 4 °C, the cells were incubated in permeabilization buffer (0.1% Triton in phosphate-buffered saline (PBS)) for 5 minutes. After incubation in blocking buffer (3% bovine serum albumin in PBS) for 1 hour, the cells were incubated with primary antibodies against DCLK1 (1:200; Abcam, Cambridge, UK; catalog #ab31074) or α-tubulin (1:1000; Sigma) overnight at 4 °C. Cells were then treated for 1 hour with secondary antibody. Alexa Fluor 568 tetramethylrhodamine isothiocyanate-conjugated donkey anti-rabbit IgG (1:500; Sigma) was used for DCLK1, and donkey anti-mouse IgG (1:500; Sigma) was used for α-tubulin. Hoechst 33342 solution diluted in PBS was used for nuclear staining. After mounting, the cells were visualized with a fluorescent microscope (Carl Zeiss). Clinical samples (DCLK1, 1:500) were stained with an automated immunostainer (Ventana XT System; Ventana Medical Systems, Tucson, AZ, USA) by using heat-induced epitope retrieval and a standard diaminobenzidin detection kit (Ventana). The sections of tumor tissue were observed under a light microscope. Two different pathologists scored the immunostained sections. The scoring of DCLK1 staining was carried out based on two different parameters: staining intensity and the amount of tissue involved. The intensity was measured and scored from 0 (no staining) to 3 (strong staining). The amount of tissue involved was measured and scored from 0 (no tissue involved) to 4 (over 80% involved). To obtain the composite score, the intensity score was multiplied by the tissue involvement score.

**Western blotting**

The expression of DCLK1 protein was detected by western blotting. After extraction of protein from each cell line and loading on 10 % sodium dodecyl sulfate–polyacrylamide gel electrophoresis, the blots were incubated overnight at 4°C with the primary antibody. We used the antibodies for DCLK1 (1:500), GFP (1:500; Abcam; catalog#Ab290) or a-tubulin (1:200; Santa Cruz Biotechnology, CA, USA: catalog#TU-02). The appropriate secondary antibodies were added for 1 h, and the protein expression was visualized with enhanced chemiluminescence (ECL) by the ECL Western blot testing detection system (GE Healthcare,Buckinghamshire, UK).
